# Clinical Unity and Community Empowerment: The Use of Smartphone Technology to Empower Community Management of Chronic Venous Ulcers through the Support of a Tertiary Unit

**DOI:** 10.1371/journal.pone.0078786

**Published:** 2013-11-12

**Authors:** Edel Marie Quinn, Mark A. Corrigan, John O’Mullane, David Murphy, Elaine A. Lehane, Patricia Leahy-Warren, Alice Coffey, Patricia McCluskey, Henry Paul Redmond, Greg J. Fulton

**Affiliations:** 1 Department of Surgery, Cork University Hospital, Cork, Ireland; 2 Department of Computer Science, University College Cork, Cork, Ireland; 3 School of Nursing and Midwifery, Brookfield Health Sciences Complex, University College Cork, Cork, Ireland; The University of Queensland, Australia

## Abstract

**Background:**

Chronic ulcers affect roughly 60,000 Irish people, at a total cost of €600,000,000, or €10,000 per patient annually. By virtue of their chronicity, these ulcers also contribute a significant burden to tertiary outpatient vascular clinics.

**Objective:**

We propose utilizing mobile phone technology to decentralise care from tertiary centres to the community, improving efficiency and patient satisfaction, while maintaining patient safety.

**Methods:**

Bespoke mobile software was developed for Apples iPhone 4 platform. This allowed for the remote collection of patient images prospectively and their transmission with clinical queries, from the primary healthcare team to the tertiary centre. Training and iPhones were provided to five public health nurses in geographically remote areas of the region. Data were uploaded securely and user end software was developed allowing the review and manipulation of images, along with two way communication between the teams. Establishing reliability, patients were reviewed clinically as well as remotely, and concordance analysed. Qualitative data were collected through focus group discussion.

**Results:**

From October to December 2011 eight patients (61–83 yrs, mean 75.3 yrs) with chronic venous ulceration and their five public health nurses were recruited. Data were transmitted using 3 G, Edge, GPRS and WiFi, at a mean speed of 69.03 kps. Concordance was 100% for wound bed assessment, 80% for skin integrity/colour and 60% for exudate assessment. Focus group analysis explored the concept, practicalities and future applications of the system.

**Conclusions:**

With an evolving national data network, the secure transmission of clinical images is a safe alternative to regular clinic appointments for patients with chronic venous ulceration. With further development, and packaged as a freely downloadable application, this has the potential to support the community care of chronic wounds.

## Introduction

Chronic leg ulcers are a significant health economic burden, estimated to cost 6.5 million euro per annum in Ireland [1] and $2.5 billion in the United States [2]. Patients with chronic venous ulcers generally have their ulcers reviewed and dressed regularly in the community by their general practitioner (GP) or public health nurse (PHN). However, they also periodically attend a hospital based dressing clinic for review by a vascular surgeon as community care under the direction of a specialist service has been shown to improve healing rates [3]. Commonly, patients with stable or healing ulcers do not have any major alterations made to their treatment plan at this clinic visit and the primary purpose is for the surgeon to see the ulcer, ensuring that healing is progressing. Marston et al [4] have shown that 25% of ulcers are not healed after 16 weeks treatment and 4% persist after 1 year’s treatment. Therefore chronic ulcers contribute a significant patient load to outpatient clinics.

A high concordance has been shown previously between direct assessment of a chronic ulcer and electronic assessment via review of a digital image of an ulcer [5]. Transmission of photographs of ulcers to a tertiary referral centre for assessment has also been shown to be both acceptable and potentially cost-saving [6], [7]. We therefore hypothesised that using a mobile smartphone application to facilitate the specialist review of a digital image of a patient’s ulcer is both safe and feasible. This in turn would reduce the number of times a patient would have to physically present to the dressing clinic for review, as proposed in a study by Salomhofer et al. [5].

The purpose of this study was to design and investigate the feasibility and safety of using a mobile application to facilitate the assessment of chronic wounds/ulcers in the community. We have shown that it is both safe and feasible to transmit clinical images for tertiary review as a safe alternative to regular clinic appointments for patients with chronic venous ulceration.

## Materials and Methods

### Pilot Digital Imaging Assessment

Prior to commencement, we established the reliability of the clinical interpretation of digitally acquired images by performing a pilot study assessing the ability of a clinical nurse specialist (CNS) in wound care to assess a digital image. This sudy was conducted over the course of 6 weeks at the wound care clinic with 35 patients studied. Each patient had their relevant wound digitally photographed using a 5 megapixel digital camera. The image was immediately uploaded to a high-resolution monitor. This image was assessed by the CNS using a standard wound care assessment sheet constructed to individually assess 7 features of the wound. Immediately following this the CNS physically assessed the patients wound and again scored the wound using the same scoring system. The scores were recorded and statistically assessed at the end of the study. The result demonstrated that a clinical assessment can be reliably made based on digital images with an overall concordance of 96% between phyiscal and digital images.

### Application Development

**Figure 1:**
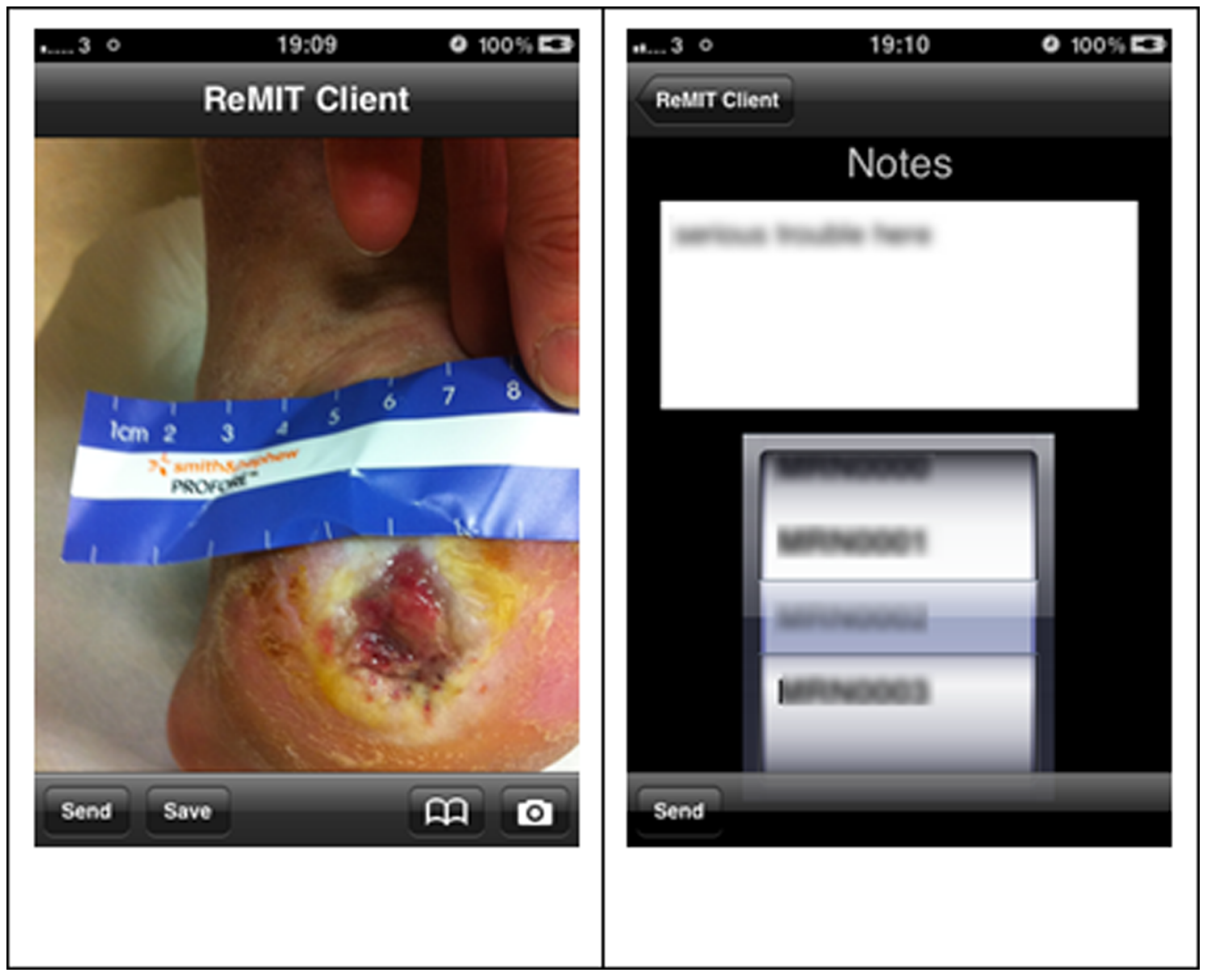
Images are captured on the iPhone as shown (A). The user then chooses the option to “send” in the lower left corner of the screen and is presented with the opportunity to add free-text notes prior to selecting the patient’s name from the list stored on the “app” (B). Once the correct name is chosen, the user presses “send” again in the lower left corner and the image and accompanying notes are sent to the tertiary centre database.

Having established consistency between digital images and clinical assessment, an iPhone application (app) was developed, entitled ReMIT Client. The ReMIT Client app was designed to be used by medical professionals without specialist training. The app presents a simple interface in which the user can select an image, either by capturing from the camera or selecting an existing image. The image along with a patient identifier and any notes are sent to the remote server when the user selects the “Send” button, or the user can save the image for later use by selecting the “Save” button (all illustrated in *Figure 1*). The user is then informed if the transfer to the server is successful.

### Image Transfer and Viewing

**Figure 2:**
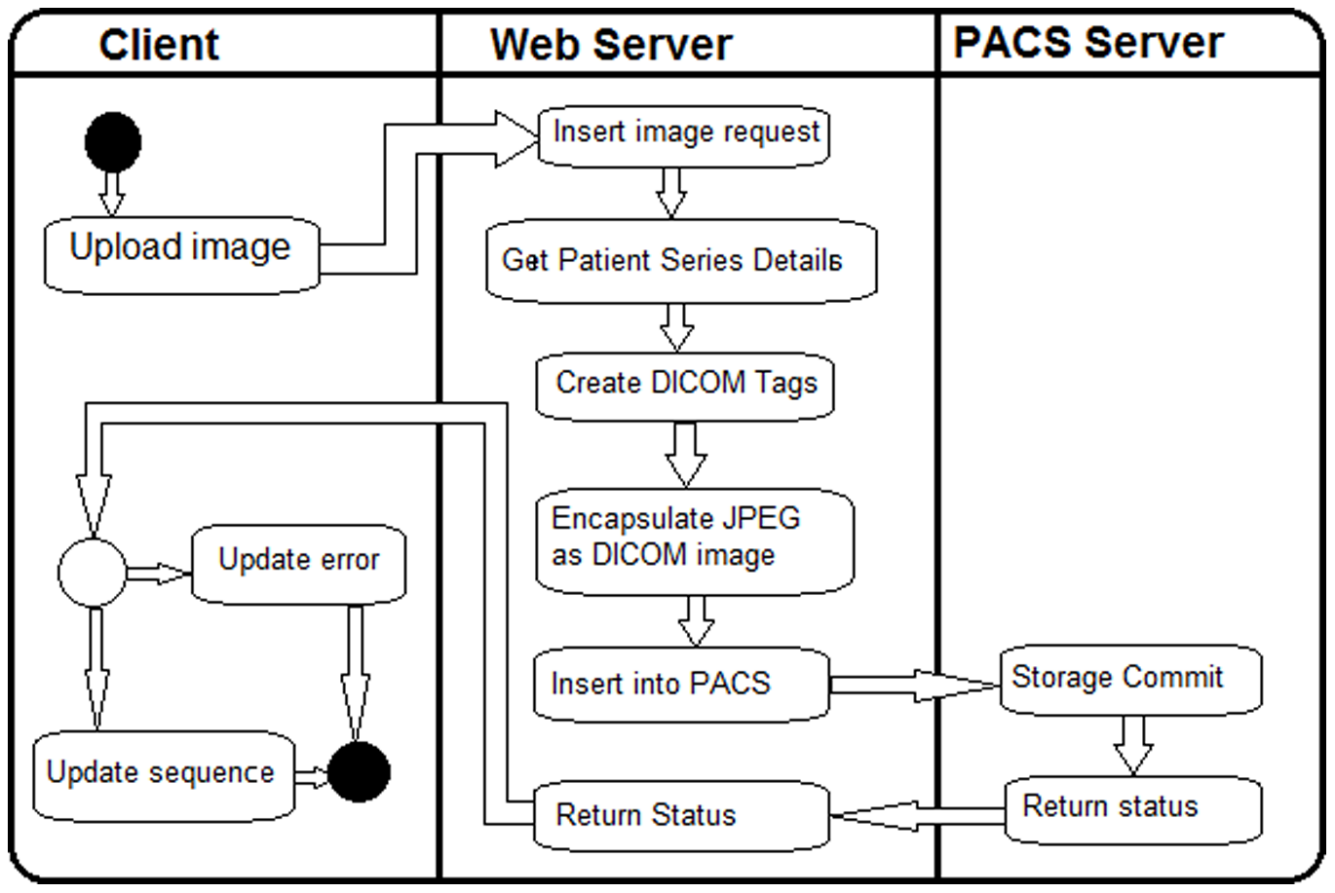
Workflow in the ReMIT system.

The ReMIT project is built around the dcm4che collection of open source applications, in particular its DICOM Image Manager/Image Archive server, dcm4chee. The dcm4chee server coupled with the image processing application OsiriX, implements a picture archiving and communications system (PACS). The client iPhone application (ReMITclient) is integrated with the PACS through the use of a web-based interface. The workflow between the ReMIT Client app, the web-based interface and the PACS server is shown in *Figure 2*.

In the ReMIT project we created a DICOM study and series for each patient. The web-based interface, combines the patient details, dynamically generated content, such as the time and date, and the image data (baseline JPEG format) into a DICOM image. This image is transferred to the PACS as an image in the DICOM series for the patient. If the transfer is not successful, usually due to network problems or by the user cancelling the transfer, the sequence is not updated and this information is reported back to the ReMIT Client, so that the user knows that they need to transfer the image again.

The consultant’s review workstation is a Macintosh computer running the medical imaging software OsiriX. Using the extensible architecture of OsiriX we developed a plugin (called REMIT) to enable the consultant access uploaded images from a PACS server. The user can examine, manipulate and annotate the images, and can review the images individually or as a chronological series of images of individual cases. The software is written as a native plugin in Objective-C and is integrated within the mixed C/C++/Objective-C environs of OsiriX. The user can examine the selected images, perform various transformations of the images including colour correction and white balancing to compensate for poor lighting and colour anomalies in the original image. The user can also access various measurement and annotation tools in the plugin toolbar directly above the image. Across the bottom of the window is a text entry field where the consultant can make comments on the particular image or image set. When the review is completed the changes and annotations are merged into a new version of the image(s), thus preserving the original images. Using this approach the consultant is able to review the case as a longitudinal study of images.

### Patient Recruitment

Following the initial pilot study and application development, 8 patients attending a University teaching hospital with chronic ulcers for more than sixteen weeks were recruited, along with their five PHNs, in a geographical area extending to 120 km from the specialist centre. PHNs in a range of geographical areas, with various levels of experience and different patient demographics were chosen to achieve a maximum variation sample. Unselected PHNs with patients regularly attending the vascular clinic were approached to consider participation and no nurse declined participation. Data were collected regarding the underlying disease process, duration of ulcer treatment, ankle-brachial index (ABI) measurements and co-morbidities.

These patients had digital images of their ulcer taken in the community on a regular basis by their PHN using an iPhone 4 (Apple, Cupertino, CA, USA) mobile Smartphone. The digital images were of the ulcer, with more than one view taken if required. These images were sent, along with information about the patient’s current dressings, directly to a secure encrypted computer database containing the patient’s medical history at the tertiary hospital. This was performed using the specifically created iPhone (Apple) application, ReMIT.

Photos were then reviewed on the specifically designed IT interface accessed through a designated terminal (Apple), by a consultant vascular surgeon or registrar (minimum postgraduate year 4 doctor). This image display software allowed the reviewing doctor to make an ulcer assessment using a standardized proforma based on the transmitted images(s). Patients continued to attend the dressing clinic at their usual intervals and the same standardized proforma assessment of their ulcer was made at their clinical assessment, again by the consultant or registrar. Ulcer assessments for each patient were not necessarily performed by the same person but were assessed immediately by an available senior doctor in line with day-to-day practice in the vascular clinic. An assessment of the image and a clinic visit assessment were made within one week of each other. Same day assessments were not possible due to the long round-trip travel to be undertaken by patients. Therefore PHNs were asked to transmit a photograph taken at their closest home assessment either immediately pre- or post-clinic assessment. The proforma recorded ulcer location, size, wound bed, colour, degree of exudate and surrounding skin condition. The proforma required the assessor to select the best of four or five options to describe the ulcer under each heading. Concordance of the assessment based on observation of the photograph versus viewing the ulcer in person was recorded; concordance was measured as agreement between both of the assessors as to which of the options best fit for each of the individual characteristics of the ulcer.

Ethical Approval for this study was granted by the Cork Teaching Hospitals Research Ethics Committee. Written informed consent was required from all patients, on the ethics committee approved consent form.

### Participant Feedback

Qualitative data were collected from the PHN’s using a combination of a focus group and semi structured interviews, where attitudes towards, satisfaction and difficulties with, and suggestions for improvement of the service were explored. Data was collected from PHNs until theoretical saturation, by virtue of recurring themes, was reached. Discussions were digitally recorded and transcribed for analysis. Thematic content analysis used to explore the data generated by the participants’ narratives.

## Results

### Quantitative Results

In the initial pilot involving 35 patients the results demonstrated that a clinical assessment can be reliably made based on digital images with an overall concordance of 96% between phyiscal and digital images.

Between October and December 2011 eight patients and their five PHNs were then recruited to the study. Patients had a mean age of 74.2±3 years, with seven males and one female. Patient demographics are summarised in *Table 1*. PHNs were practicing predominantly in catchment areas requiring long travel distances to the tertiary referral hospital (8.2–114 km) (*Table 2*).

**Table 1:** Patient demographics and clinical data.

	Results
**Age (mean)**	74.2 years ±3.0 years (61–83 years)
**Gender**	7 Male: 1 Female
**Distance from Hospital (mean) (range)**	72.1 km ±11.7 km (5.2–114 km)
**Ankle Brachial Index (mean) (range)**	1.05 (1.00–1.10)*
**Arterial Disease (% with)**	16.7%
**Venous Disease (% with)**	100%
**Diabetes (% with)**	16.7%
**Traumatic Ulcer (%)**	16.7%
**Duration of ulcer(s) (mean)**	8.8 years ±4.3 years (2–30 years)

* Mean ankle brachial indices were all greater than 1 despite one patient having arterial disease; this was due to this same patient also having diabetes mellitus.

**Table 2:** Public Health Nurse (PHN) areas of practice within Ireland and distance from tertiary referral centre.

	Distance to tertiary hospital(one way)
**PHN 1– Cork City**	8.2 km
**PHN 2– Schull, Co Cork**	103 km
**PHN 3– Bantry, Co Cork**	81.9 km
**PHN 4– West Cork Area**	78.8 km
**PHN 5– Tralee, Co Kerry**	114 km

Each patient was assessed with two patients being assessed twice, a number of months apart, during the study time-frame (total ten assessments). Clinic and photograph assessments were generally concordant; there was concordant clinic and photograph assessment of the wound bed in 100% of cases, peri-wound skin integrity and colour assessments were concordant in 80%, exudate assessment concordance was lowest at 60%. The size of the ulcer was often written in the text accompanying the picture sent by the PHNs and so concordance was not assessed for size assessment. The picture quality was assessed as adequate or better in 80% of cases.

Mean image upload times and speed were 290.86 seconds and 63.09 kbps (range 15–2394 seconds, 1.64–331.99 kbps). Image upload success rates were 37% (75/204 total image upload attempts), indicating that in the majority of cases it took more than one attempt to forward an image from the community to the tertiary referral centre, in keeping with the slow data upload speeds encountered at times in remote areas.

### Qualitative Results

In general, participant PHN feedback was overwhelmingly positive in terms of a ‘proof of concept’ and future potential of the iPhone application as illustrated by the quotations in *Table 3*.

**Table 3:** Public Health Nurse (PHN) reaction to the project.

*“Personally I think it’s a fantastic idea,… so I’m all for it, I think it’s a great idea”*
*“The idea is brilliant”*
*“I would say that my feedback on it, is that it is hugely beneficial, it is a very positive, fantastic concept. Bits to be ironed out but that is like anything else and you have to try them to find out. I think the potential of it is really huge.”*
*“I think the potentiala of it is really huge, I really do.”*
*“I would say it would be a positive [the app], yeah it probably just needs to be refined and you know I would say it was a positive step”*

Progressing from the general overview provided by participants, two key themes were apparent from transcript analysis. These included; (1) Access and communication pathway potential and (2) ‘App’ usability.

1. **Access and communication.** Potential for enhanced access to tertiary centre expertise and improved communication pathways was a predominant theme in the findings of this study. Facilitation of direct access by the ‘app’ to the tertiary referral centre and therefore consultant expertise for decision-making support in relation to wound care was a central issue raised by all participants;

“*Particularly I would feel being in [a rural area] where we actually don’t have any vascular service,… that I suppose a great benefit for me would be to talk directly to the consultant that you want to see, you know the person that you would like to refer the patient too…direct communication with them*”

“..*it gives us access to the centre which we wouldn’t have otherwise, so I’m all for it, I think it’s a great idea*”

In relation to “proper access and proper referrals”, the issue of current communication pathways or referral systems which exist and their inadequacy was highlighted as being a significant impediment to efficient and effective treatment of wounds in the community;

*“…. And I’m actually working as a clinical nurse specialist, so the patients that I actually see would be the ones that the public health nurses are having the problems with, say the complex cases that are not healing and are not going along the normal, you know progressing in the normal way, and the problem for me is that sometimes I’m having to go through a GP then to refer them on somewhere else ….so you have a lot of delays…., [with this] it’s not going through several people”*

Difficulties were evident however in receiving feedback about the photographic images during the pilot from the tertiary centre which was a frustration for the participants;

*“I think going forward we just need to tighten up the interaction between us and the hospital… if you want to move things on in patient care going forward, it’s the vascular team we would really want to engage with”*

It was highlighted that the facilitation of such direct communication between tertiary and primary care professionals would have the potential to positively impact on outpatient clinics and as a result improve patients experience of their wound care management;

*“There is a move on to move things away from acute services, to stop the pressure on acute services all the time and to maybe use primary care, make tertiary clinics better. I think this [the app] would enhance that”*

*“You could cut back on the repeat reviews [in outpatient clinics] if we could do it in the community, quite often, we know where we are going with it and quite often they have a repeat appointment and they go off any way. It’s just clogging up your clinics”*

*“There is a huge interest in it, the trial, as one of our patients is travelling two and a half hours…and then they might have to wait a couple of hours at the clinic all day…he actually told me he goes home and goes to bed for two days [afterwards]”*

1. **‘App’ usability.** This theme encompasses the categories of ‘app’ user-friendliness and operation practicalities and supportive ‘app’ technology. The majority of participants agreed that the application and iPhone were “simple”, “familiar” and “straight-forward” to use with only minimal instruction required to become comfortable with the system;

“*There is definitely tweaking to do, but you are talking twenty minutes of someone showing you what to do tops and you would have no problem using it really. I suppose lots of people are familiar with iPhones anyway”*

One of the participants who was not familiar with iPhone technology reported that while the system was not complicated to use, additional troubleshooting support if required would be reassuring;

*“…. I found that the first time I was a bit slow with it the first morning when I was trying to send a picture. It took me awhile to do it. It’s not that complicated, but I suppose, what I felt was,..it could initially be a problem in the beginning but once you’ve got the hang of it, its probably ok….”*

In terms of the application, it was acknowledged as being user-friendly and having excellent photographic quality sufficient for assessment purposes. A number of refinements to enhance its usability were also suggested. These included upgrading the application’s ability to store information in a manner whereby each patient could have a separate named folder, entries be registered in date sequence, and a log of sent items received by the tertiary centre be kept and fed back to the sender;

*“..so what you actually need … is that each patient on the app would have a folder within the phone itself and that would be dated”*

*“The other thing that I thought,….it’s only afterwards that you would think of this, that you probably would need to label one, two, three and four,…the second time round I think I [would] put the dates on as well because at one stage this man had cellulitis and he ended up cancelling the first appointment and going into hospital but I was able to take a picture. I took the picture of the leg when he actually had the cellulitis and then it was maybe six weeks later, we remade the appointment and I was able to tell them he had IV treatment……and you could even see the difference in those pictures between the leg when he’s had cellulitis and six weeks post cellulitis after IV antibiotics”*

In terms of the technology supporting the application, that is, the iPhone and Wi-Fi network, issues relating to the durability of the iPhone and immediate connectivity to a network for uploading photos were raised. In relation to durability, it was suggested that an ‘iPad’ rather than an ‘iPhone’ would be more durable given the conditions that the hardware would be subjected to. It would also have the added advantage of being more user-friendly in terms of having a larger screen and keyboard for inputting information than an ‘iPhone’;

*“The only problem I had with the iPhone is the battery time on it …and it [needs to be] a much tougher thing, it’s going to be slapped around from my dressing table in the car, the houses, anywhere, the iPhones tends to be a lot more delicate. And the battery time was a bugbear with me. I hadn’t got the time to be plugging it in everyday, I hadn’t always got an office to plug it in.I’ve got shared office space, with two or three other disciplines, it’s my reality”*

*“With an iPad it would be sturdier …you can get proper cases for it”*

In relation to Wi-Fi access required to send the photographic images to the tertiary centre for assessment, connectivity was an issue depending on the geographical area of the patient. Slow internet connections and image upload failures were a source of frustration for participants. To resolve this issue a number of participants used their personal internet connections or the Wi-Fi access at their work bases;

*“The problem would be there isn't Wi-Fi where we are [the clinics]. I have tried it everywhere and the only way was to put my own Wi-Fi on the iPhone and to use that to send”*

*“Try and try to send as you can be sitting there for 20–30 minutes & you don’t know if it is gone”*

“*the coverage there [at patient’s home] wasn’t great so as I took the picture I could never to send it straight away, it would never go, I’d often wait until I got back to my base and I found that they were going when I sent them from there, there was better coverage”*

The issues highlighted above in terms of durability, user-friendliness and network coverage are important to consider in ensuring that healthcare professionals’ workload is not adversely impacted upon.

## Discussion

Our study has shown that mobile technology can be used to help assess chronic wounds and is safe and reliable with a high concordance of assessment of wound bed, periwound skin integrity and colour. Other studies have previously demonstrated photographic assessment to be a reliable means of assessment [5], [8] and our study supports this and demonstrates that Smartphone technology can be safely used to empower community practitioners in the care of chronic wounds. We used a 5 megapixel iPhone camera with good image quality results (only 20% less than adequate, including the learning curve at the beginning of the pilot).

The five PHNs in our study universally embraced the concept of our study showing it to be a potentially well-received initiative were it to be commenced across the catchment area. The main advantages to its use were seen to be facilitation of direct access to specialist expertise, improved communication pathways, enhanced and greater care in the community with resultant reduced patient travel and ease of use of the system. Due to the fact that such direct communication and access does not exist within the current primary to tertiary centre referral structure, the participants had forged indirect and informal access pathways to clinical expertise which was not seen as satisfactory. The facilitation of such direct communication between tertiary and primary care professionals was highlighted as having the potential to positively impact on outpatient clinics and as a result improve patients’ experience of their wound care management. It was emphasized in this pilot study that such support could be improved by the use of referral protocols and ‘buy in’ information sessions.

Use of photography as a component of wound assessment pathways is emerging as an area of increased interest over the last number of years. Chanussot-Deprez showed that photography in rural areas can be used to allow adequate wound management with advice from distant centres while saving on travel costs for patients and the health system [9]. Khan et al used digital photography to follow pin sites from external fixators in 5 patients over a year [10]. In this study patients self-photographed their wound sites and emailed them to the treating physicians and all found this a highly satisfactory way of reducing outpatient visits. Conversely, Walker et al found that of 50 emergency department patients with facial lacerations asked to follow-up by forwarding a self-taken digital photograph of their wound, no patient complied with forwarding of the photograph [11]. This suggests that leaving the responsibility for photography in the hands of patients would not be a reliable means and hence the need for PHNs or other responsible health professional, as used in our study.

Hill et al have shown that videoconferencing is a useful tool for obtaining ulcer evaluation close to that of a home visit [12]. However, videoconferencing requires both parties to be available at the same time to assess the ulcer. Photographic assessment allows for non-emergency assessment to take place in the community at a time suitable for the PHN, with review at a time convenient for the tertiary centre, and without a need to link schedules.

Al-Hadithy et al have described how Smartphones are being increasingly used by medical professionals [13] and are used increasingly to photograph wounds. Smartphones therefore represent a familiar tool for implementation of an information technology system for wound review. A recent study of smartphone use amongst trainee doctors in the UK showed that 74.8% of junior doctors own a smartphone and they are frequently used for support and to enhance clinical care [14]. Indeed one of our PHNs commented on how “lots of people are familiar with iPhones anyway”.

Specific areas requiring improvement were also identified. Feedback from the tertiary referral centre was found to be slow at the introduction of the pilot. While the community side of the study used mobile technology to forward photographs for review, the receiving side of the study used static technology on a single computer terminal. This required review of images from a single fixed point. Enhanced feedback could be potentially improved using mobile technology within the tertiary referral centre to enhance rapid feedback to the PHNs. Such technology was not available at the onset of this study, however several viable options now exist.

A number of technological changes to the ‘app’ have been suggested, including ability to store information in the application in separate folders for each patient with photographs timed and dated. Additionally the confirmation of receipt message should be stored in the sender’s application so they can confirm receipt of the image at a later time if required. These suggestions require technical adaptations to our prototype ‘app’ as it currently stands.

One of the major barriers to implementation of the concept is the lack of a reliable network signal in certain geographical regions, as evidenced by the variable upload times and recurrent failed attempts to upload images to the database. The majority of PHNs could use networks at their work bases to send images but even then found the upload time to be slow at times. Repeated sending of images may also incur a greater cost from the internet service provider. A future development in the ‘app’ could include reduction in the size of the images captured to allow easier transmission. However effects this may have on image quality would need to be evaluated further.

The small sample size limits the empirical evidence that can be drawn from this pilot study. However, this was not the primary aim of the study, which was to assess the implementation phase, feasibility and end-user feedback in relation to the system. Similarly, having ulcer assessments performed a number of days apart may lead to slight discrepancies in the appearance of the ulcers on assessment days. However, as all ulcers were chronic in nature, each having been present for at least sixteen weeks, it was felt that the speed of healing would be unlikely to reach significant levels within a number of days.

The positive feedback from this pilot study is very encouraging for implementation of the concept at a wider level in the community. Factors previously identified by others to influence successful health information technology implementations [15] include a) technical factors – the heavy workload, frequently limited computer skills and frequent turnover of staff require systems that are easy to learn and easy to use, [16] our ‘app’ has been shown to be easy to use with minimal training time required for five PHNs which should allow for smoother introduction; b) organisational factors – this system has been shown to be easily integrated into the community setting within the PHNs own daily practice, it may however require some alterations to fit in with organisational practice at the tertiary referral centre by making this end of the concept mobile also; and c) social factors – it is necessary to encourage potential users to “ buy in” to the new system. Ensuring successful implementation amongst a group of new users has been shown to be best achieved through use of a “clinical champion” who leads introduction of the system into practice [15]. Mahmoud et al also describe how a champion is a critical factor in propelling health IT implementation efforts [16]. Box et al successfully used clinical champions to encourage participation in the implementation of an electronic record keeping system for cardiac catheterization laboratory procedures [17]. Clinical champions were users of the system involved in feedback and changes to ensure optimizations of the system [17]. Our recruited PHNs have shown enthusiasm for the project and would represent potential clinical champions for implementation of the project at a wider level. The key to motivating staff to partake is demonstrating the adoption of technology as a tool to assist them in achieving their goal of improving the quality of patient care [16]. This study has identified clear potential improvements in the quality of care through the implementation of this system.

One of the major benefits for patients in implementing this system would be removal of the need to travel long distances for specialist input into ulcer care. This would have cost saving implications for both patients and their carers. A number of patients with chronic ulcers are long-stay patients in community hospitals or nursing homes [18] and therefore require ambulance transport to the tertiary centre. Reduction in transport frequency would also have reductions in cost implications for regional ambulance services. Finally, reduction in patient attendances at tertiary referral clinics would increase available slots for other patients, thus contributing to a reduction in waiting lists for specialist services, and therefore improve the ratio of new to return patients.

### Conclusions

Digital image assessment is a safe and reliable means of assessing chronic non-healing ulcers in the community. High quality digital images can be securely sent by PHNs via a specifically designed Smartphone application for review at a tertiary referral centre many kilometers away from the patient’s home location. PHNs find this application to be easy-to-use, improves communication with the tertiary referral centre and allows convenient, easily accessible ulcer care in the community. With some minor adjustments to the piloted system developed by this study, this application could be used across the community to reduce patient attendances at vascular outpatient clinics whilst still maintaining active tertiary specialist input to their care.
